# Exploring the Molecular Mechanisms of Autism Induced by Early Childhood Exposure to Bisphenol A Based on Network Toxicology and Molecular Docking

**DOI:** 10.3390/ijms27146317

**Published:** 2026-07-16

**Authors:** Guanmin Zheng, Liyu Wang, Guoqiang Wang, Yuhang Zhang, Xue Han, Guo Qiu, Yangang Sun, Lanying Pei, Suhui Wu, Hanbing Li

**Affiliations:** 1 School of Medicine, Henan University of Chinese Medicine, Zhengzhou 450046, China; 2 Academy of Chinese Medical Sciences, Henan University of Chinese Medicine, Zhengzhou 450046, China; 3 College of Veterinary Medicine, Henan Agricultural University, Zhengzhou 450046, China

**Keywords:** network toxicology, bisphenol A, autism spectrum disorder, molecular docking, molecular dynamics simulation

## Abstract

Early childhood exposure to bisphenol A (BPA) is closely associated with autism spectrum disorder (ASD), though the precise molecular mechanisms remain unclear. To investigate this, we employed an integrated computational approach combining network toxicology, molecular docking, and molecular dynamics simulation. Potential targets of BPA and ASD-related genes were collected from multiple databases, identifying 57 overlapping targets. Protein–protein interaction network analysis highlighted 16 core targets among them. Gene Ontology and KEGG pathway enrichment analyses indicated these targets are primarily involved in synaptic transmission, GABAergic signaling, and neuroactive ligand–receptor interactions. Molecular docking demonstrated potential binding between BPA and several core targets, including estrogen receptor 1 (ESR1), gamma-aminobutyric acid type A receptor subunit beta-2 (GABRB2), and amyloid precursor protein (APP), with binding energies below −5 kcal/mol. The stability of the BPA-ESR1 complex was further supported through 100 ns molecular dynamics simulation. These results suggest that BPA may contribute to ASD by disrupting neuroendocrine pathways and synaptic function via interactions with key targets such as ESR1.

## 1. Introduction

Autism spectrum disorder (ASD) is a complex neurodevelopmental disorder, the incidence of which is on the rise globally, posing a serious public health challenge. Data officially released by the World Health Organization (WHO) indicate that the prevalence of ASD has been gradually increasing in recent years, with approximately one child in every hundred affected [1]. ASD is widely recognized as a multifactorial disorder resulting from interactions between genetic vulnerabilities and environmental exposures. While heritability estimates are high, no single genetic cause accounts for a majority of cases, and emerging evidence supports that environmental factors, including endocrine-disrupting chemicals such as Bisphenol A (BPA), contribute to disease risk by modifying gene expression or interacting with specific genetic variants [2]. BPA is an organic compound with two hydroxyphenyl groups, commonly used as an additive in the production of epoxy resins and polycarbonate plastics. It is one of the most widely manufactured chemicals in the world. BPA is extensively found in everyday products, such as baby bottles, food packaging materials, medical devices and thermal printing paper [3,4]. Humans can be passively exposed to BPA through the oral, respiratory and dermal routes [5], and it tends to accumulate over time [6]. Biomonitoring studies conducted in the United States, Germany and Canada have detected BPA in the urine of over 90% of the population [7]. The U.S. Centers for Disease Control and Prevention tested 2500 urine samples from the general population and found that BPA was detectable in as high as 92.6% of individuals, with children having significantly higher BPA exposure levels (4.5 ng/mL) compared to adults (2.5 ng/mL) [8]. Accumulating evidence suggests that BPA is an emerging endocrine-disrupting chemical (EDC) closely associated with the development of cardiovascular diseases, reproductive dysfunction, and even cancer. Its lipophilic nature allows it to cross the blood–brain barrier, posing a serious threat to the nervous system [9], especially to the reproductive, immune and neurological systems of children [10,11,12,13,14,15]. Multiple epidemiological studies have shown a strong association between maternal or infant exposure to BPA and severe neurological disorders in children, such as anxiety, depression, aggressiveness, ASD, attention deficit/hyperactivity disorder and cognitive impairments [16,17,18,19].

Similarly, studies have indicated that prenatal exposure to BPA is related to ASD-associated behaviors and abnormal brain development in males [20]. Notably, in three case–control studies, serum BPA levels in children with ASD were significantly higher than those in healthy controls [21,22,23]. Additionally, research has found that juvenile exposure to BPA induces abnormal emotional and social behaviors in mice, which are associated with hyperactivity in the basolateral amygdala (BLA) region. The abnormal discharge in the BLA region is related to emotional disturbances and social impairments [24]. However, the specific molecular mechanisms underlying ASD induction by early childhood exposure to BPA have not yet been reported. This information is crucial for balancing the benefits of BPA in children’s food safety and other applications against its potential health risks, so as to effectively safeguard public health and environmental safety.

Network toxicology is a toxicological research method based on the concept of systems biology. It constructs biological molecular networks to analyze the interference and impact of chemical substances or environmental pollutants on these networks within the body, thereby revealing their toxic mechanisms [25]. Molecular docking can predict the binding modes of ligands and their affinity with receptor proteins. It can also calculate the binding energy of the interaction between a toxin and its target. The lower the binding energy, the more potential the toxin-target conformation, and the higher potential for their interaction. This study will systematically characterize the molecular mechanisms through which BPA potentially induces ASD, and identify potential therapeutic targets using molecular docking techniques. This will enrich the environmental etiology theory of ASD and provide a new theoretical basis for the prevention and treatment of ASD. Furthermore, this research will provide scientific evidence for assessing the health risks of BPA, and offer reference for formulating policies and regulations to protect children’s health.

## 2. Results

### 2.1. BPA Toxicity Prediction Results

Using the ProTox-3.0 platform and admetSAR 3.0 software, we predicted that BPA can exert toxic effects on the blood–brain barrier (BBB), with prediction probabilities of 53% and 64%, respectively (Table 1). Based on this result, this study hypothesizes that BPA may induce neurotoxicity by disrupting the BBB and subsequently entering the brain tissue.

### 2.2. Identification of Potential Targets of BPA and ASD and Intersection Targets

**Figure 1:**
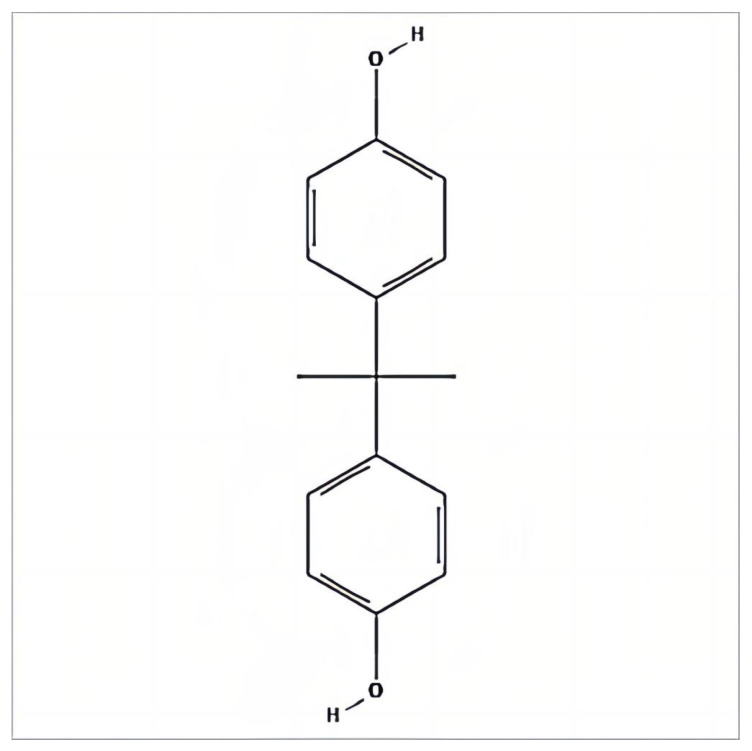
The 2D structure of BPA.

**Figure 2:**
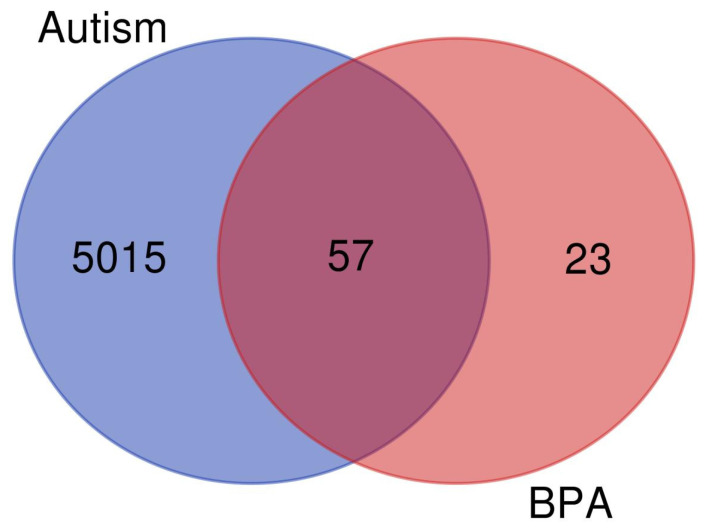
Venn diagram of BPA and ASD targets.

First, using the 2D structure of BPA (Figure 1), 34 potential targets were identified through the SwissTargetPrediction platform, 42 through the TCMIP platform and 16 through the Similarity Ensemble Approach (SEA) database. A total of 92 potential BPA targets were collected, and after removing duplicates, 80 unique targets were obtained. For ASD-related disease targets, the top 5000 targets were selected from the GeneCards database, 12 from the Therapeutic target database (TTD) and 97 from the Online mendelian inheritance in man (OMIM) database, among the top 5000 target genes in the GeneCards database, the lowest Relevance score was 2.052010536, indicating that all of these genes are fundamentally associated with ASD. A total of 5109 disease targets were collected, and after removing duplicates, 5072 unique targets were obtained. Finally, the intersection of these results yielded 57 potential targets for BPA-induced ASD (Figure 2).

### 2.3. Establishment of Protein-Protein Interaction (PPI) Network and Determination of Core Targets

**Figure 3:**
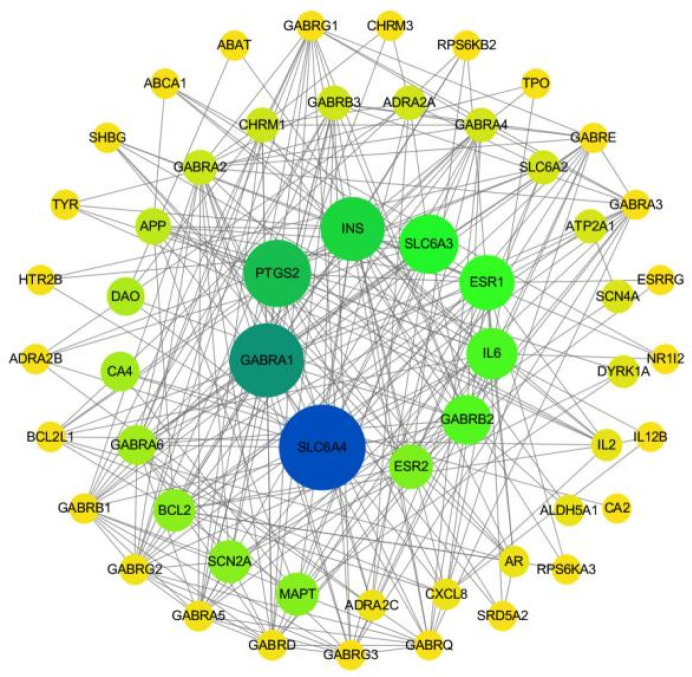
PPI network diagram. Each edge represents an interaction between proteins, and each node represents a target. The size and color of the circular nodes are proportional to the BC values, with blue indicating higher values and yellow indicating lower values.

**Figure 4:**
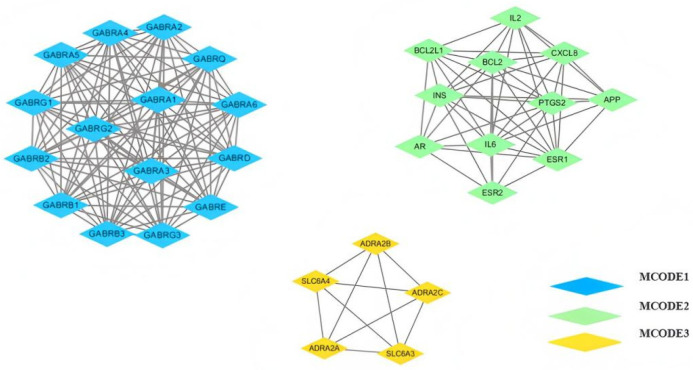
Clustering results obtained using MCODE. The blue network represents MCODE1, the green network represents MCODE2, and the yellow network represents MCODE3.

**Figure 5:**
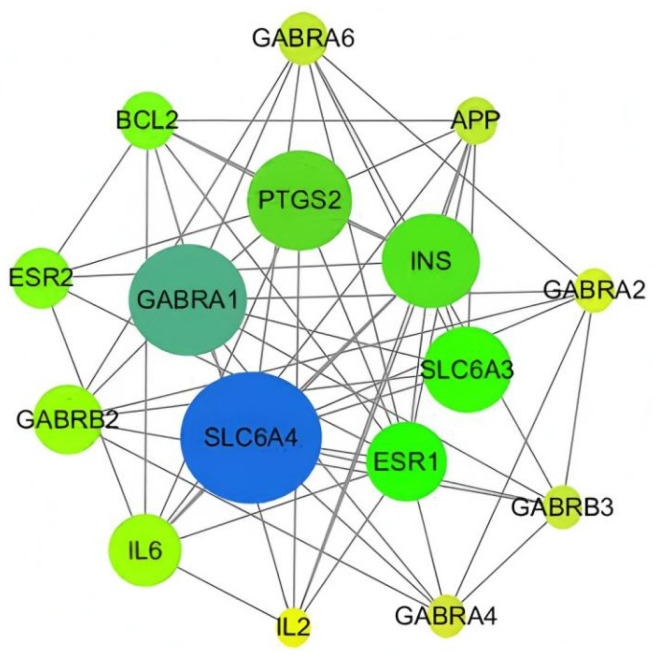
Core target network diagram. The size and color of the circles are proportional to the BC values, with blue indicating higher values and yellow indicating lower values.

The PPI network diagram of ASD induced by BPA was obtained using the STRING database (Figure 3). The MCODE plugin in Cytoscape was used to analyze the 57 intersection targets, and 3 sub-networks were identified (Figure 4). Based on the core target screening criteria specified in the methods (Betweenness centrality (BC) > 0.00536911, Closeness centrality (CC) > 0.40601504, Degree value > 9), a total of 16 core targets associated with BPA-induced ASD were obtained (Table 2). To more clearly demonstrate the interactions between these core targets, a core target network diagram was constructed (Figure 5).

### 2.4. GO Analysis

**Figure 6:**
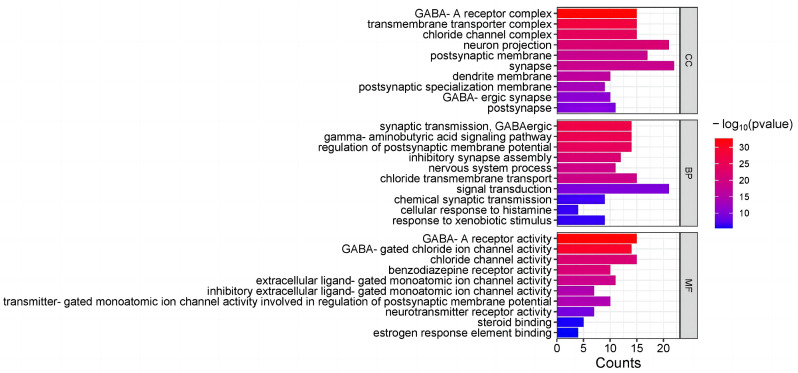
Results of GO enrichment analysis for the intersecting genes of BPA and ASD. The histogram shows the top 10 entries for BP, CC, and MF sorted by adjusted *p*-value, which reflects the statistical significance of the enrichment. Red indicates a smaller *p*-value and higher enrichment significance, while blue indicates a larger *p*-value and lower significance. The length of each bar corresponds to the number of genes, reflecting the magnitude of enrichment in the corresponding category.

GO functional enrichment analysis of the intersection targets yielded 232 significant entries (150 BP, 35 CC, 47 MF), from which 30 entries are summarized (Figure 6). The specific enriched terms provide crucial insights into the potential neurotoxic mechanisms of BPA.

GABA-related terms showed pronounced enrichment across all three GO categories: “GABA-A receptor complex” in cellular components, “GABAergic synaptic transmission” in biological processes, and “GABA-A receptor activity” in molecular functions. This strongly suggests that BPA exposure may primarily disrupt inhibitory neurotransmission. This finding is particularly significant given the well-established role of GABAergic dysfunction in neurodevelopmental disorders.

Furthermore, the enrichment of the term “regulation of postsynaptic membrane potential” indicates BPA’s potential to interfere with fundamental neuronal signaling processes, while the involvement of chloride channel complexes suggests possible disturbances in ionic homeostasis that is essential for normal neural function. The convergence of these findings across multiple functional categories underscores that BPA may disrupt the development and function of coordinated neural circuits by targeting key components of the GABAergic system.

### 2.5. KEGG Analysis

**Figure 7:**
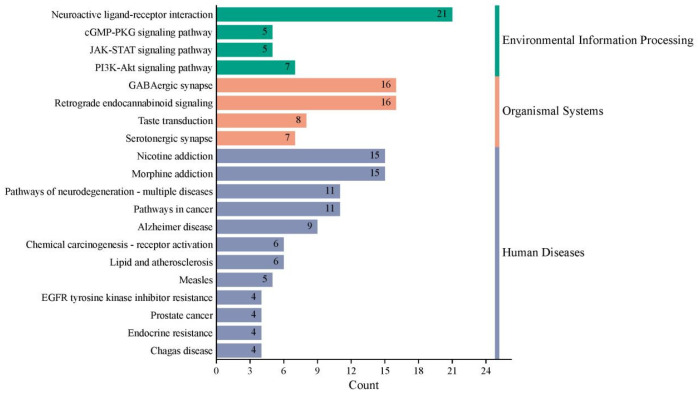
Results of KEGG enrichment analysis performed on the intersecting genes of BPA and ASD. The bar chart visualizes the top 20 enriched KEGG signaling pathways and primarily presents these pathways along with the number of genes enriched in each. Green, orange and blue indicate the KEGG categories Environmental Information Processing, Organismal Systems and Human Diseases, respectively. Bar length corresponds to the number of enriched genes: longer bars represent a greater number of enriched genes, while shorter bars represent fewer. The numbers annotated on each bar also indicate the number of enriched genes in that pathway.

KEGG pathway enrichment analysis revealed distinct patterns of pathway involvement across different functional categories. The targets were distributed among several major categories: Environmental Information Processing contained the neuroactive ligand–receptor interaction pathway (21 genes), Organismal Systems encompassed both the GABAergic synapse pathway and retrograde endocannabinoid signaling pathway (16 genes each), and Human Diseases included nicotine addiction and morphine addiction pathways (15 genes each) (Figure 7).

**Figure 8:**
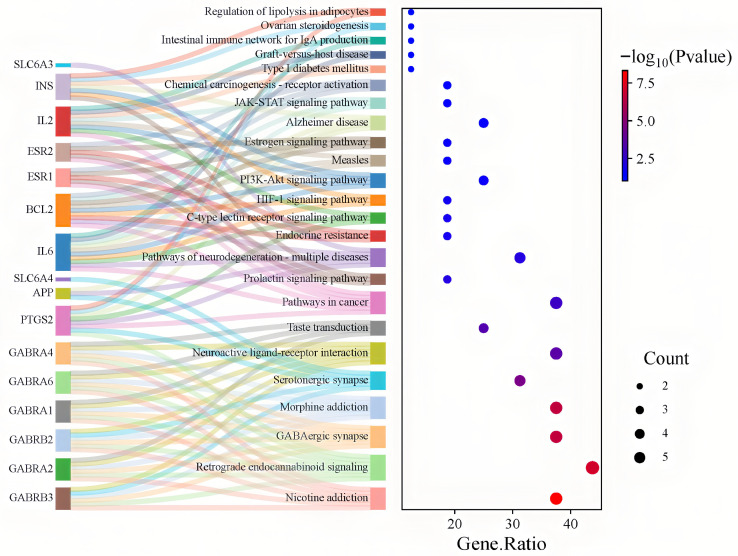
Results of KEGG enrichment analysis for core targets. The left side displays the associations between genes and their corresponding pathways. The dot size indicates the number of enriched genes, and the dot color represents the *p*-value, where a redder dot corresponds to a lower *p*-value.

Further KEGG enrichment analysis of the core targets yielded 24 results. The pathways with the highest number of enriched genes were retrograde endocannabinoid signaling, neuroactive ligand–receptor interaction, and nicotine addiction (Figure 8). These three pathways demonstrated the highest gene representation among all analyzed core targets.

Beyond this categorical distribution, the specific pathway enrichment results provide mechanistic insights into BPA’s potential neurotoxicity. The robust enrichment of neuroactive ligand–receptor interactions suggests BPA may disrupt fundamental neuronal communication systems, while the concurrent involvement of GABAergic synapse pathways indicates specific targeting of inhibitory neurotransmission. The identification of retrograde endocannabinoid signaling is particularly noteworthy, as this system plays crucial roles in synaptic plasticity and neural circuit modulation. Furthermore, the association with addiction-related pathways may reflect BPA’s potential impact on reward circuitry and reinforcement mechanisms. Collectively, these findings indicate that BPA-induced neurodevelopmental effects likely involve coordinated disruption across multiple signaling systems, rather than effects limited to isolated pathways.

### 2.6. Molecular Docking Results

**Figure 9:**
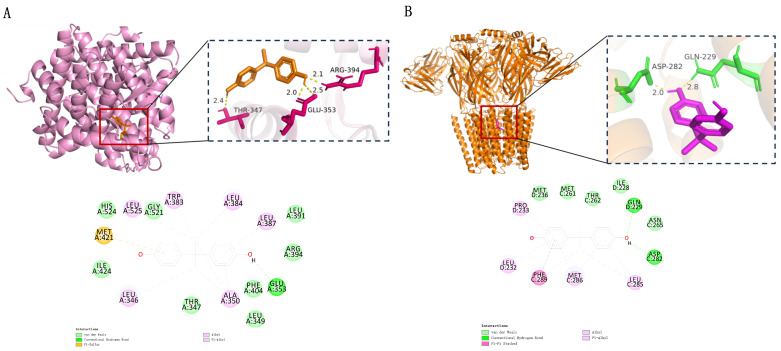

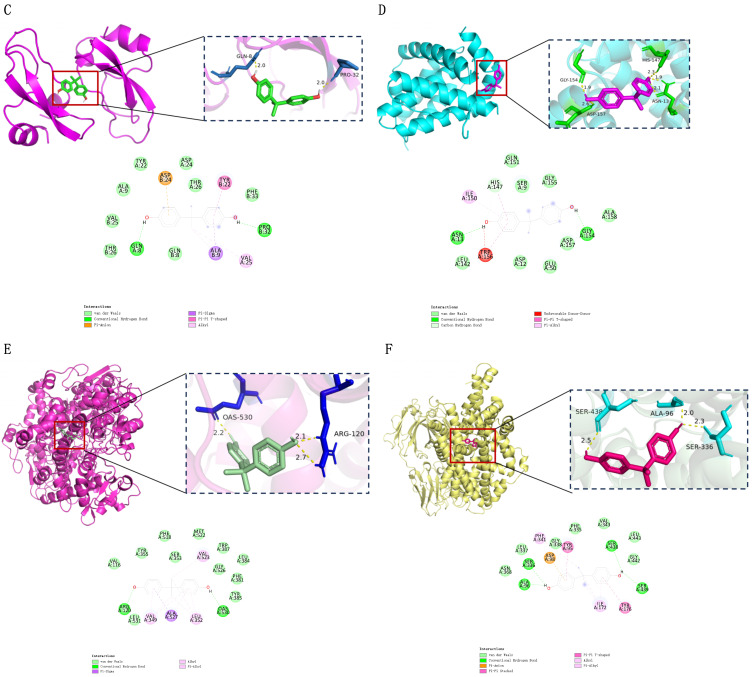
Molecular docking diagrams of BPA binding to core targets. Panels (**A**–**F**) show the molecular docking results of BPA with ESR1, GABRB2, APP, BCL2, PTGS2 and 5-HTT, respectively.

Molecular docking was performed for six core targets: Estrogen receptor 1 (ESR1), Gamma-aminobutyric acid type A receptor subunit beta-2 (GABRB2), Amyloid precursor protein (APP), B-cell lymphoma 2 (BCL2), Prostaglandin-endoperoxide synthase 2 (PTGS2) and 5-hydroxytryptamine transporter (5-HTT) (Figure 9). The binding energies of BPA with all these proteins were less than −5 kcal/mol, indicating that all the formed complexes adopt conformations conducive to binding (Table 3). Notably, ESR1 exhibited the strongest binding affinity at −6.22 kcal/mol. The specific interactions include hydrogen bonds, hydrophobic interactions and π-stacking interactions (Table 4).

Critically, our docking results provide mechanistic insights into how BPA may contribute to ASD pathogenesis. The strong binding to ESR1 suggests BPA can act as an endocrine disruptor. This is supported by experimental evidence confirming the BPA-ESR1 interaction, which can dysregulate estrogen-sensitive genes crucial for neurodevelopment and synaptic plasticity [26,27]. The stable binding to GABRB2, a key GABA-A receptor subunit, indicates potential direct disruption of inhibitory neurotransmission, which aligns with the established role of GABAergic signaling deficits in ASD [28]. Furthermore, the interactions with 5-HTT, APP, PTGS2 and BCL2 suggest that BPA could additionally impair serotonin reuptake, promote neuroinflammatory pathways, and disrupt neuronal survival, respectively. All these mechanisms are proposed to underlie ASD-related molecular and behavioral abnormalities.

In summary, our predictions indicate plausible mechanisms by which BPA may disrupt neuroendocrine signaling, synaptic function and neuronal homeostasis, thereby potentially increasing ASD risk.

### 2.7. Molecular Dynamics Simulation Results

**Figure 10:**
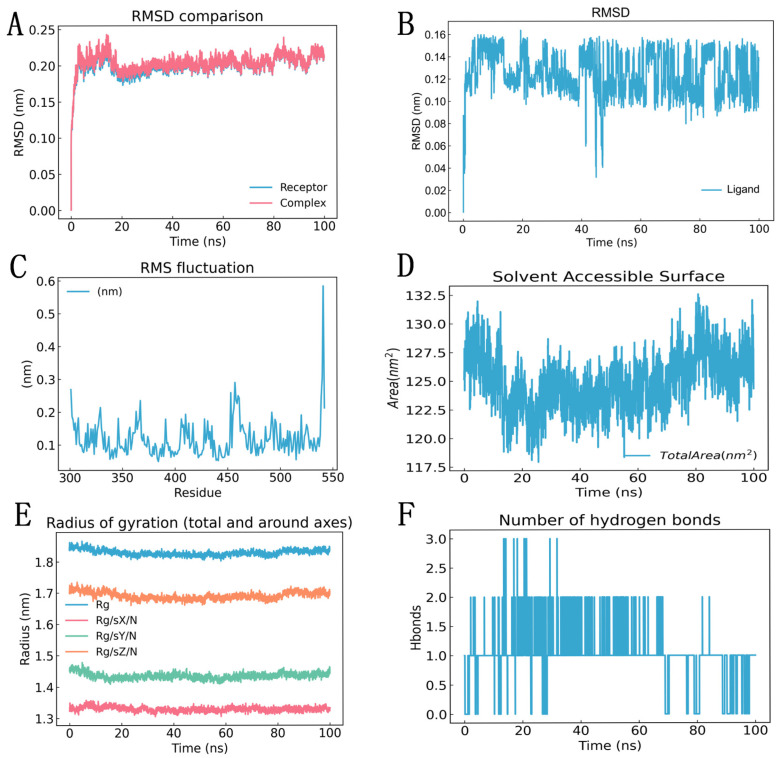
Results of Molecular Dynamics Simulation. (**A**) RMSD results of ESR1 and BPA-ESR1; (**B**) RMSD results of BPA; (**C**) RMSF results of ESR1; (**D**) SASA results of BPA-ESR1; (**E**) Rg results of BPA-ESR1; (**F**) Number of hydrogen bonds results of BPA-ESR1.

To validate the stability of the BPA-ESR1 complex from molecular docking, a 100 ns molecular dynamics simulation was performed. The system reached equilibrium early in the simulation: Root mean square deviation (RMSD) of protein backbone and complex stabilized after 1.2 ns (Figure 10A), and RMSD of ligand converged to 1.3 Å after 0.69 ns (Figure 10B), indicating a stable binding mode. The root mean square fluctuation (RMSF) results showed no fluctuations > 1 nm (Figure 10C), indicating that the BPA-ESR1 complex is highly stable. A stable solvent-accessible surface area (SASA) and Radius of gyration (Rg) further indicated structural compactness upon BPA binding (Figure 10D,E). Persistent hydrogen bonding throughout the simulation confirmed sustained molecular recognition (Figure 10F). These results support a structurally stable BPA-ESR1 complex, suggesting a plausible mechanism by which BPA may disrupt estrogen signaling and contribute to neurodevelopmental alterations relevant to ASD.

## 3. Discussion

BPA is a widely used additive and manufacturing material for food packaging, and has extensive applications in daily human life. Clarifying its toxicological effects helps people better prevent and avoid its adverse health impacts. In this study, 57 potential targets associated with BPA-induced ASD were screened via Venn diagram analysis. Subsequently, 16 core targets related to BPA-induced ASD were identified using a PPI network. GO and KEGG functional enrichment analyses of these core targets revealed a close association between BPA exposure and synaptic transmission. Six core targets were selected for molecular docking, and the results showed that all six core target proteins have the potential to bind to BPA. ESR1, which had the lowest binding energy, was selected for molecular dynamics simulation. The simulation results confirmed the stable binding between ESR1 and BPA, with the overall system remaining stable and compact. This indicates that the ESR1 target protein may play a critical role in the pathogenesis of BPA-induced ASD.

ESR1 is a member of the nuclear receptor superfamily and participates in the regulation of multiple signaling pathways in the central nervous system. ESR1 directly binds to the estrogen response element (ERE) of the *BDNF* gene to promote BDNF expression [29], which further activates downstream TrkB receptors and their corresponding signaling pathways (PI3K/Akt, MAPK/ERK) [30]. Abnormalities in the BDNF signaling pathway are one of the core pathological features of ASD [31], so the ESR1-BDNF-MAPK pathway is possibly associated with synaptic development dysregulation in ASD.

In addition, existing studies suggest that regulation of postsynaptic membrane potential and amino acid-related synaptic mechanisms may act as intermediate pathways affecting ASD development [32,33], which shows some consistency with the GO functional enrichment results obtained in this study. Notably, previous research has confirmed that BPA can directly bind to ESR1 [27], which aligns with the molecular docking and molecular dynamics simulation results of this study. BPA can directly interfere with ESR1 function, which may affect synaptic formation and function in offspring, ultimately leading to learning and memory impairments and abnormal social behaviors [34]. Studies have also found that BPA exposure can increase DNA methylation at the *ESR1* gene promoter in the hippocampus of offspring rats, thereby inhibiting ESR1 expression. This may disrupt neuroplasticity as well as brain development and function, eventually leading to ASD-related behavioral phenotypes [34]. Therefore, this study speculates that early childhood exposure to BPA may also induce ASD by directly interfering with ESR1 function or affecting ESR1 expression, leading to neuronal plasticity damage. The core targets identified in this study, including *ESR1*, *GABRB2*, and *SLC6A4*, may harbor functional genetic variants that could potentially alter protein structure, expression, or binding properties. For instance, some studies suggest that polymorphisms in *ESR1* might modify endocrine effects associated with BPA exposure [35]. GABRB2 is a key regulatory factor in the pathogenesis of ASD and is closely associated with the GABAergic signaling pathway [28]. When *GABRB2* gene mutations or epigenetic modifications (such as DNA hypermethylation in the promoter region) lead to downregulated expression of this gene, chloride ion influx mediated by GABA receptors decreases, which weakens inhibitory postsynaptic potential (IPSP). This inhibitory neurotransmission defect disrupts the brain’s excitation/inhibition (E/I) balance, leading to excessive neuronal excitation and abnormal synaptic plasticity. Dysfunction of this signaling pathway can further trigger downstream overactivation of the mTOR pathway, dysregulated BDNF expression, and microglia-mediated neuroinflammation, all of which contribute to the core symptoms of ASD: social impairments, repetitive behaviors, and sensory abnormalities [36,37,38]. Relevant studies have found that in ASD model mice, impaired GABAergic neurotransmission is mediated by a presynaptic mechanism, which is accompanied by reduced GABA release and decreased expression of GABA-a and GABA-b receptor subunits [39]. Another study also reached the conclusion based on experiments in ASD model mice that ASD-like behaviors are caused by impaired generation of GABAergic interneurons [40]. These findings show some consistency with the GO functional and KEGG pathway enrichment analysis results obtained in this study. Therefore, we speculate that BPA binding to the GABRB2 protein may inhibit its activity, disrupt the normal function of the GABAergic signaling pathway, and ultimately induce ASD. While our molecular dynamics simulation focused on ESR1 due to its well-characterized interaction with BPA, the importance of GABRB2 in mediating potential BPA effects on GABAergic transmission is acknowledged. Future computational and experimental studies are warranted to further explore the dynamics of BPA-GABRB2 binding.

APP is a transmembrane glycoprotein that plays an important role in various physiological processes, including cell signaling, neuronal development, synaptic plasticity, and cell adhesion. BPA exposure can lead to oxidative stress and neuroinflammation, which in turn affect the metabolism and function of APP [41]. Abnormal cleavage of APP produces β-amyloid (Aβ), which triggers neuroinflammation and synaptic toxicity through activation of the NF-κB pathway [42], leading to the pathological features of ASD. Therefore, this study speculates that BPA may also induce ASD by directly binding to APP and altering its normal metabolism, ultimately resulting in neuroinflammation.

5-HTT dysfunction leads to imbalances in 5-HT levels, a condition commonly observed in ASD patients. Studies have found that BPA exposure can cause epigenetic modifications in the promoter region of the *5-HTT* gene, such as increased DNA methylation levels, thereby inhibiting 5-HTT expression [43]. This downregulation may reduce 5-HT reuptake, further altering 5-HT concentration in the synaptic cleft [44]. Additionally, BPA may affect the efficiency of 5-HT transport by binding to the 5-HTT protein or changing its conformation.

PTGS2, also known as COX-2, is a key enzyme that catalyzes the conversion of arachidonic acid (AA) to prostaglandins (such as PGE2). PGE2 exerts multiple physiological functions in the nervous system, including regulating neuroinflammation, synaptic plasticity, and neuronal activity [45]. In ASD, PGE2 levels are significantly elevated, which may be associated with COX-2 overactivation [46]. Studies have found that BPA can activate the MAPK pathway, particularly the phosphorylation of ERK1/2, thereby inducing COX-2 expression [47]. Therefore, this study speculates that BPA binding to COX-2 may activate its enzymatic activity, increasing PGE2 levels in the nervous system and thereby inducing ASD.

BCL2 is an important anti-apoptotic protein that promotes neuronal survival and differentiation by inhibiting apoptosis, which is crucial for normal brain development [48]. In the brains of individuals with autism, BCL2 expression is significantly reduced, and the expression and phosphorylation/activation of Akt, the upstream regulator of BCL2, are also significantly decreased. This downregulation of Akt may result from reduced BDNF concentrations. Cumulative evidence suggests that downregulation of the BDNF-Akt-Bcl2 anti-apoptotic signaling pathway in the brains of individuals with autism may be one of the potential causes underlying ASD pathogenesis [49]. Therefore, it is speculated that BPA binding to BCL2 may inhibit its activity, exacerbate apoptosis and neuronal damage, and thereby induce ASD.

In summary, this integrated computational study employed network toxicology, molecular docking, and molecular dynamics simulations to investigate the potential mechanisms of BPA-induced ASD. Our analysis identified 57 common targets and screened 16 core targets through PPI network analysis. GO and KEGG enrichment analyses suggested these targets are potentially involved in synaptic transmission and GABAergic signaling pathways. Molecular docking results indicated potential binding between BPA and the six core targets, with the strongest relative binding affinity observed for ESR1. This finding was further supported by molecular dynamics simulations. These findings suggest that BPA may contribute to ASD pathogenesis by disrupting neuroendocrine pathways and synaptic function. While this work provides valuable insights and testable hypotheses, this study utilized the top 5000 target genes from the GeneCards database for ASD target gene prediction. This broad threshold may encompass genes with weak supporting evidence, indirect associations, or nonspecificity. These predictions are derived solely from computational models and require further experimental validation to confirm their biological significance. Nevertheless, this study offers new insights into the molecular mechanisms of BPA-induced ASD in early childhood, which not only helps reveal the neurotoxic mechanisms of BPA and identifies potential targets for early intervention, but also provides a scientific basis for guiding public health policies, such as strengthening regulation of food packaging materials for children.

## 4. Materials and Methods

### 4.1. Prediction of BPA Toxicity

Toxicity prediction of BPA was conducted using both the ProTox-3.0 platform (https://tox.charite.de/protox3/) (accessed on 17 February 2025) and the admetSAR 3.0 platform (https://lmmd.ecust.edu.cn/admetsar3/index.php) (accessed on 17 February 2025). The ProTox-3.0 platform was selected for its comprehensive prediction of various organ toxicities (such as hepatotoxicity, carcinogenicity and immunotoxicity) and its provision of a predicted median lethal dose (LD_50_), enabling a holistic toxicity risk assessment. In contrast, admetSAR 3.0 is a full-coverage absorption, distribution, metabolism, excretion and toxicity (ADMET) platform that offers a wide range of endpoints and rapid predictions. The use of these two complementary platforms allowed for cross-validation of neurological toxicity concerns and provided a broader perspective on the potential toxicological profile of BPA. First, the SMILES sequence of BPA was retrieved from the PubChem database (https://pubchem.ncbi.nlm.nih.gov/) (accessed on 17 February 2025). The SMILES sequence was then input into the “Prediction” module of the aforementioned databases, and the prediction results were downloaded.

### 4.2. Prediction of BPA Targets

First, the term “BPA” was searched in the PubChem database to find the best match and verify the correctness of the name and molecular formula. The SMILES sequence was then submitted to the Swiss Target Prediction website (http://www.swisstargetprediction.ch/) (accessed on 19 February 2025), with the species set as “Homo sapiens” and the prediction condition probability set to >0. The prediction results were downloaded, and targets with probabilities >0 and <0.1 were selected. These targets were considered as potential targets of BPA. Additionally, the SDF file of BPA was uploaded to the TCMIP platform (http://www.tcmip.cn/TCMIP/index.php/Home/Login/login.html) (accessed on 19 February 2025), with a similarity threshold set at ≥0.6. The prediction results were downloaded and considered as BPA targets. Furthermore, the SMILES sequence was input into the SEA database (https://sea.bkslab.org/) (accessed on 19 February 2025), and the prediction results were downloaded. To standardize the nomenclature of the targets, the UniProt database (https://www.uniprot.org/) (accessed on 19 February 2025) was used. Finally, the prediction results from all sources were merged, and duplicate targets were removed to obtain the final list of BPA targets.

### 4.3. Prediction of ASD Targets

ASD-related targets were retrieved from the GeneCards (https://www.genecards.org/) (accessed on 21 February 2025), TTD (https://db.idrblab.net/ttd/) (accessed on 21 February 2025), and OMIM (https://www.omim.org/) (accessed on 21 February 2025) databases. The keyword “Autism” was used to search for ASD-associated targets in these databases. While these are comprehensive databases, they were selected for this study because they represent authoritative and widely utilized resources that integrate extensively curated genetic information from multiple sources, including specialized ASD repositories such as Simons Foundation Autism Research Initiative (SFARI) Gene. This approach ensures the acquisition of a broad and reliable collection of potentially relevant targets, which is a established practice in network pharmacology and toxicology studies. For the GeneCards database, which yields a large number of predicted targets, the top 5000 targets with the highest relevance scores for ASD were selected [50]. The predicted targets from all three databases were subsequently merged, and duplicates were removed to establish the final disease target library.

### 4.4. Identification of Intersection Targets Between BPA and ASD

The intersection targets between BPA and ASD were identified using the Venn website (http://bioinformatics.psb.ugent.be/webtools/Venn/) (accessed on 23 February 2025). The BPA target library and the ASD target library were submitted to the website to obtain the intersection targets.

### 4.5. Construction of PPI Network and Identification of Core Targets

The PPI network was constructed using the STRING database. The genes of the intersection targets between BPA and ASD were input into the STRING database, with the species set as “Homo sapiens”. The “minimum required interaction score” was set to “medium confidence > 0.4” for analysis. The results generated by STRING were then imported into Cytoscape software version 3.7.1. Cytoscape is a powerful network biology tool that can calculate topological properties of each node in the network, including degree, closeness centrality, betweenness centrality and average shortest path length [51]. The analysis was performed using the “Tools-Network Analyze-Network Analysis-Analyze Network” function, and the results were exported as a CSV file for core target screening. Core targets were selected based on the following criteria: BC > median, CC > median, and Degree value > median, where the median values were calculated from the entire network of 57 intersecting targets. BC measures the number of shortest paths passing through a node; CC is the reciprocal of the average distance to other nodes; and degree value measures the number of direct connections to other nodes. The final core targets were obtained by combining the results.

### 4.6. GO Function and KEGG Pathway Analysis

GO function and KEGG pathway analysis were primarily conducted using the DAVID database (https://david.ncifcrf.gov/) (accessed on 10 March 2025). GO analysis evaluated biological processes (BPs), molecular functions (MFs) and cellular components (CCs). The KEGG database was selected for its comprehensive coverage of metabolic and signaling pathways, standardized annotations and wide application in toxicological studies [52,53]. The intersection targets from the Venn diagram were uploaded to the DAVID database, with the species set as human. The top 10 GO categories and top 20 KEGG pathways were selected based on the smallest to largest *p*-values. Additionally, KEGG enrichment analysis was performed on the core targets using the DAVID database to further elucidate the pathways involved in ASD. Finally, the results of the GO and KEGG analyses were visualized using the WeiShengXin website (http://www.bioinformatics.com.cn/) (accessed on 10 March 2025).

### 4.7. Molecular Docking

Molecular docking was used to predict the binding modes and affinities between BPA and the core target proteins. First, the 3D structure SDF file of BPA was obtained from the PubChem database and converted to a PDB file using OpenBabelGUI software v3.1.1. The 3D structures of the target proteins were obtained from the RCSB Protein Data Bank (PDB; https://www.rcsb.org/) (accessed on 10 April 2025) with the following PDB codes: ESR1 (5DXE), GABRB2 (6 × 3T), APP (1AAP), BCL2 (5FCG), PTGS2 (5F19), and SLC6A4 (6AWO). Water molecules and original ligands were removed from the target proteins using Pymol v2.5.0. The proteins were then imported into AutoDock v4.2.6 for hydrogenation, charge calculation and nonpolar hydrogen merging. For each target protein, the spatial extent of its binding pocket was defined based on the spatial positions of co-crystallized ligands in the corresponding PDB structures. Specifically, AutoDock v4.2.6 was employed to read PDB files and position the docking grid box at the geometric center of the co-crystallized ligands within each target protein’s PDB structure. The central coordinates and dimensions of the grid boxes for each target protein are as follows: ESR1 (11.011 Å, 2.534 Å, 17.102 Å) (67 Å, 67 Å, 79 Å); GABRB2 (138.048 Å, 136.293 Å, 132.249 Å) (119 Å, 139 Å, 125 Å); APP (16.316 Å, 18.972 Å, 36.522 Å) (91 Å, 121 Å, 119 Å); BCL2 (75.198 Å, 181.072 Å, 2.905 Å) (125 Å, 125 Å, 111 Å); PTGS2 (22.594 Å, 40.999 Å, 39.560 Å) (79 Å, 75 Å, 111 Å); SLC6A4 (−35.811 Å, −14.454 Å, 17.363 Å) (75 Å, 75 Å, 115 Å). Genetic algorithm parameters were set for 10 runs, with 10 docking models generated for each protein–ligand pair. The population size was set to 1.5025×10^9^ maximum evaluations, with a mutation rate of 0.02 and a crossover rate of 0.8. The model with the lowest binding energy was selected as the final result and visualized using Pymol v2.5.0. In molecular docking, a lower binding energy generally indicates a more favorable predicted binding affinity, suggesting a higher potential for interaction between the ligand and the receptor. However, docking scores are approximate and should not be interpreted as direct evidence of conformational stability or biological activity. Based on established criteria in molecular docking studies, a binding energy threshold of −5.0 kcal/mol was employed to identify significant binding interactions, as this value represents the typical threshold for predicting potential biological activity in small molecule–protein interactions. A binding energy less than −5.0 kcal/mol indicates higher potential binding of the ligand to the receptor [54].

### 4.8. Molecular Dynamics Simulation

Molecular dynamics simulation is a powerful computational technique that simulates the dynamic behavior of molecular systems to predict their physical and chemical properties. This study used molecular dynamics methods to investigate the interaction mechanisms between BPA and ASD-related targets in detail. This technique allows observation of molecular interactions at the atomic level, including bond formation and cleavage, as well as molecular rotation and translation, thereby elucidating how BPA affects the structure and function of target proteins. The Gromacs software package v2026.1, a widely recognized molecular simulation tool, was used for detailed experiments. It provides comprehensive tools for building, energy minimizing, equilibrating and simulating the dynamic behavior of molecular systems [55]. Among the core targets, ESR1 was selected for molecular dynamics simulation because it exhibited the lowest binding energy (−6.22 kcal/mol) and is the most extensively characterized target of BPA with known molecular mechanisms [56]. Based on the molecular docking results from the previous step, the optimal binding conformation of BPA with the target protein predicted by AutoDock v4.2.6 was used as the initial conformation for molecular dynamics simulation. The Gromacs software package v2026.1 was used to perform 100 ns of molecular dynamics simulation. The Amber19SB force field was used for protein processing. The optimal point charge (OPC) water model was used to solvate the system, and a periodic boundary with a water box of 1.0 nm was established. The particle mesh Ewald (PME) method was used to calculate long-range electrostatic interactions, and the Monte Carlo ion placement method was used to introduce an appropriate number of sodium and chloride ions to neutralize the system’s charge. Before the formal simulation, system energy minimization and equilibration were performed in three steps: (1) energy minimization of each system using 10,000 steps of the steepest descent algorithm and 10,000 steps of the Conjugate Gradient algorithm; (2) pre-equilibration of each system for 1,000,000 steps with a time step of 2 fs, maintaining constant particle number, volume and temperature (300 K); (3) pre-equilibration of the entire system for 1,000,000 steps with a time step of 2 fs, maintaining constant particle number, pressure (1 atmosphere), and temperature (300 K). After system energy minimization and equilibration, 100 ns of molecular dynamics simulation was performed without any constraints, with a time step of 2 fs.

## Figures and Tables

**Table 1:** Predicted toxicity outcomes of BPA.

Classification	Predicted Probability		Predicted Results
	ProTox-3.0	admetSAR 3.0	
Blood–brain barrier	53%	64%	Active
Respiratory toxicity	21%	13.2%	Inactive
Nephrotoxicity	20%	41%	Inactive
Carcinogenicity	10%	39.6%	Inactive
Mutagenicity	2%	16,1%	Inactive

**Table 2:** Information on core target genes.

Name	BC	CC	Degree Value
*APP*	0.02588081	0.46153846	11
*IL6*	0.08248748	0.49541284	17
*ESR1*	0.09752234	0.5	18
*INS*	0.13106957	0.51923077	20
*SLC6A4*	0.21529469	0.53465347	14
*SLC6A3*	0.11274904	0.51923077	11
*PTGS2*	0.14413819	0.54	14
*GABRB2*	0.07645967	0.46956522	19
*IL2*	0.00785758	0.42519685	10
*BCL2*	0.04846938	0.45	14
*ESR2*	0.05568414	0.45	12
*GABRA4*	0.01605164	0.44628099	16
*GABRA6*	0.03776111	0.48214286	18
*GABRA2*	0.02025889	0.46153846	17
*GABRA1*	0.16993457	0.52941176	20
*GABRB3*	0.01801747	0.45762712	16

**Table 3:** Predicted binding affinities from molecular docking.

Ingredient	Gene	Target	Uniprot	Binding Energy (kcal·mol^−1^)
BPA	*ESR1*	ESR1	P03372	−6.22
BPA	*GABRB2*	GABRB2	P47870	−6.20
BPA	*APP*	APP	P05067	−5.89
BPA	*BCL2*	BCL2	P10415	−5.76
BPA	*PTGS2*	PTGS2	P35354	−5.74
BPA	*SLC6A4*	5-HTT	P31645	−5.66

**Table 4:** Detailed information of molecular docking.

Name	Hydrogen Bond	Hydrophobic Interaction	π-Stacking
BPA-ESR1	GLU 353A	LEU 387A, LEU 525A, LEU 346A, ALA 350A, TRP 383A, LEU 384A	MET 421A
BPA-GABRB2	GLN 229D, ASP 282C	LEU 232D, LEU 285C, MET 286C, PRO 233D, PHE 289C	PHE 289C
BPA-APP	GLN 8A, PRO 32B	ALA 9B, VAL 25A	TYR 22B
BPA-BCL2	ASN 13A, GLY 154A	HIS 147A, ILE 150A	HIS 147A, TRP 156A
BPA-PTGS2	ARG 120A, OAS 530A	ALA 527A, VAL 523A, VAL 349A, LEU 352A	
BPA-5-HTT	SER 336A, ALA 96A, SER 438A, SER 439A	PHE 341A, ILE 172A	TYR 95A, TYR 176A

## Data Availability

The original contributions presented in this study are included in the article. Further inquiries can be directed to the corresponding authors.
